# NIPAT as Non-Invasive Prenatal Paternity Testing Using a Panel of 861 SNVs

**DOI:** 10.3390/genes14020312

**Published:** 2023-01-25

**Authors:** Riccardo Giannico, Luca Forlani, Valentina Andrioletti, Ettore Cotroneo, Andrea Termine, Carlo Fabrizio, Raffaella Cascella, Luca Salvaderi, Pasquale Linarello, Debora Varrone, Laura Gigante, Emiliano Giardina

**Affiliations:** 1 Eurofins Genoma Group, 00138 Rome, Italy; 2 Data Science Unit, Santa Lucia Foundation IRCCS, 00179 Rome, Italy; 3 Department of Biomedicine and Prevention, Tor Vergata University, 00133 Rome, Italy; 4 Department of Biomedical Sciences, Catholic University Our Lady of Good Counsel, 1000 Tirana, Albania; 5 Genomic Medicine Laboratory-UILDM, Santa Lucia Foundation IRCCS, 00179 Rome, Italy

**Keywords:** paternity test, NGS, cffDNA, NIPAT

## Abstract

In 1997, it was discovered that maternal plasma contains Cell-Free Fetal DNA (cffDNA). cffDNA has been investigated as a source of DNA for non-invasive prenatal testing for fetal pathologies, as well as for non-invasive paternity testing. While the advent of Next Generation Sequencing (NGS) led to the routine use of Non-Invasive Prenatal Screening (NIPT or NIPS), few data are available regarding the reliability and reproducibility of Non-Invasive Prenatal Paternity Testing (NIPPT or NIPAT). Here, we present a non-invasive prenatal paternity test (NIPAT) analyzing 861 Single Nucleotide Variants (SNV) from cffDNA through NGS technology. The test, validated on more than 900 meiosis samples, generated log(CPI)(Combined Paternity Index) values for designated fathers ranging from +34 to +85, whereas log(CPI) values calculated for unrelated individuals were below −150. This study suggests that NIPAT can be used with high accuracy in real cases.

## 1. Introduction

To date, diagnostic genetic testing of the fetus during early pregnancy requires invasive procedures such as Chorionic Villus Sampling (CVS) and amniocentesis (also called amnio) associated with miscarriage risk. In 1997, it was discovered that maternal plasma contains cell-free fetal DNA (cffDNA) [1]. Most cffDNA comes from villous cells, with its concentration increasing proportionally with gestational age, enabling the chance to obtain fetal genetic information from maternal plasma. Fetal cfDNA has an average length of 150 bp (Base Pair), and comprises fragments that are shorter on average than maternal cell-free DNA. It is released by apoptotic cells in trophoblasts. Placental trophoblasts and fetuses develop from the same blastocyst and therefore share the same genome, promoting the utility of cfDNA to test fetal DNA. The placenta releases significant levels of fetal DNA into the maternal circulation, with concentrations of fetal DNA in maternal plasma showing levels of 10–20% between 10 and 20 weeks of gestation. It is well known that circulating cffDNA has a mean half-life of 16.3 minutes (min) and is undetectable in maternal plasma 2 hours post-delivery, indicating that cffDNA testing cannot be affected by carryover from previous pregnancies [2,3]. 

The advent of Next Generation Sequencing (NGS) technology, and therefore the ability to analyze sources of DNA, led to the development of several prenatal genetic tests proposed as Non-Invasive Prenatal Screening (NIPT or NIPS) [4]. The main advantage of using cffDNA is the non-invasive nature of the test compared to traditional procedures. NIPT is currently being conducted globally, with more than 10 million tests having been performed in 2018, and many countries are already using NIPT in their routine [5]. Since then, cffDNA has also been investigated as a source of fetal DNA for Non-Invasive Prenatal Paternity Testing (NIPPT or NIPAT). In particular, parental assessment is one of the central aspects of forensic genetics [6,7]. These analyses are performed by using genetic biomarkers characterized by high variability. The first genetic biomarkers to be used for human paternity testing were Short Tandem Repeats (STRs) [8]. In addition, a new class of genetic biomarkers, which can be used for parental assessment and various forensic applications, are Single Nucleotide Polymorphisms (SNPs). Compared with STR loci, SNP sites have a lower mutation rate and the amplification products of single SNP sites can be very short, which makes SNPs suitable for the analysis of highly degraded forensic samples [9]. Moreover, SNPs are related to multiple phenotypes, such as skin color, eye color, hair color, ethnicity information and susceptibility to multifactorial disorders [10,11,12,13]. 

Although many non-invasive tests have been developed so far, few data are available regarding the reliability and reproducibility of these methods [14,15,16]. In this article, we present a Non-Invasive Prenatal Paternity Test (NIPAT) analyzing 861 Single Nucleotide Variants (SNV) from cffDNA through Ion S5 NGS technology. The technology selected is already used in many laboratories for forensic next generation sequencing protocols and several commercially available kits have been validated [17,18]. The test was validated on more than 900 meiosis samples. NIPAT generated log(CPI) (Combined Paternity Index) values for designated fathers ranging from + 34 to + 85, whereas log(CPI) values calculated for unrelated individuals were below −150. Finally, the performance of NIPAT was fairly concordant with paternity compatibility threshold log(CPI) > +4, and paternity exclusion threshold log(CPI) < −4 suggested by the reference guidelines and reference literature for SNV approaches [19].

## 2. Materials and Methods

### 2.1. Selection of Samples and NGS Analysis

The samples were recruited from the molecular genetic laboratory Eurofins Genoma and signed informed consent was obtained from all of the participants before blood sample collection. Peripheral blood samples (10 mL) were collected from nine pregnant women during the first trimester of pregnancy. Buccal swabs or peripheral blood samples were collected from their partners [20]. In particular, maternal peripheral blood samples were used for the extraction of cell-free fetal DNA and subsequently employed to perform GeneSafe^®^. Maternal genomic DNA was employed to perform GeneScreen^®^ testing. GeneSafe^®^ is a non-invasive prenatal test, based on NGS technology, that allows the identification of pathogenic/likely pathogenic variants involved in inherited and de novo single-gene disorders. On the other hand, GeneScreen^®^ is a carrier screening test performed by targeted sequencing. These genetic tests were employed for the sample selection of the nine “mother – designated father” sample couples; only samples couples indicating fetuses with pathogenic/likely pathogenic variants transmitted by the father were selected for the genetic confirmation of parental relationship. 

Maternal plasma was separated from the peripheral blood by centrifugation at 1600 RCF (Relative Centrifugal Force) with a temperature of 4 °C for 10 min. Subsequently, the supernatant was transferred to a new tube and it was centrifuged for an additional 10 min at 16,000 RCF with a temperature of 4 °C.

cffDNA was extracted again using a QIAsymphony^®^ DSP Circulating DNA Kit (QIAGEN, Hilden, Germany) and QIAsymphony Automatic Extraction System (QIAGEN, Hilden, Germany) according to the manufacturer’s instructions. The QIAsymphony^®^ DSP Circulating DNA Kit is based on magnetic-particle technology for the automated isolation and purification of human circulating cffDNA. Furthermore, the QIAsymphony DSP circulating DNA Kit is a ready-to-use system for the qualitative purification of human circulating cell-free DNA from human plasma. Genomic DNA (gDNA) from paternal samples was extracted with a Qiagen DNA Mini Kit. A custom PCR amplification panel was designed through Thermo Fisher Ion Ampliseq Designer (www.ampliseq.com, accessed on 6 July 2021) using a set of 861 SNVs well-documented on dbSNP [21]. 

Single nucleotide variant selection was based on the following criteria: all SNVs had to have available population genetics data from dbSNP, 1000 genomes and/or FrogKB databases. We excluded variants labeled as indel (insertion and deletion), Multiple Nucleotide Variants (MNV)/complexes, and those that were pathogenic or likely pathogenic, and we excluded variants in highly repeated regions or in pseudogenes. The resulting SNVs were selected due to having MAF (Minor Allele Frequency) > 0.3 in at least one population, and/or were manually selected to optimize the chance of discriminating between populations to be spread across all the chromosomes (chr). In total, 638 of the 861 selected single nucleotide variants were biallelic and 223 were triallelic in the dbSNP database. The average MAF of the panel excluding triallelic SNVs was 0.321 (median in 0.347) and the number of SNVs per chromosome ranged from 12 to 95, with an average of 36. In particular, the numbers of SNVs for each chromosome were: chr1:41, chr2:61, chr3:36, chr4:95, chr5:34, chr6:60; chr7:25, chr8:74, chr9:33, chr10:26, chr11:35, chr12:33, chr13:24, chr14:31, chr15:33, chr16:35, chr17:39, chr18:23, chr19:12, chr20:29, chr21:17, chr22:17, chrX:23 and chrY:25.

The extracted cffDNA from the maternal plasma samples, as well as gDNA from the paternal samples, have been parallelly used for library preparation using the Ion AmpliSeq™ Library Kit PLUS (Thermo Fisher Scientific, Foster City, CA, USA). This kit is engineered for the rapid preparation of amplicon libraries. The Ion AmpliSeq™ Library Kit Plus is an on-plate format to facilitate sample processing, traceability and compatibility with automation. The Ion AmpliSeq™ Library Kit Plus provides high, uniform, reliable and reproducible output. Sequencing maternal cffDNA samples requires much more read depth compared to paternal gDNA samples because the evaluation of the presence of low-frequency alleles in cffDNA samples is necessary to determine the fetal genotype. For this reason, the last step of the library preparation is crucial to balance correctly maternal and paternal samples in the same pool. In fact, in order to pool cffDNA and gDNA amplified samples in order to obtain the proper number of reads, it is very important to load them 20:1 (in terms of nanograms), respectively. The entire pool is then quantified, diluted to 100 pM (parts per million), and finally processed with an Ion Chef™ Instrument for the templating and enrichment procedures. In particular, the Ion Chef™ System reduces sources of user-introduced variability and supports sequencing preparation for the Ion S5™ System. A final 500 flows sequencing has been performed using the Ion 540™ Chip running on the Ion S5™ System (Thermo Fisher Scientific, Foster City, CA, USA). The Ion S5 System is a semiconductor system which allows different sequencing workflows.

The Thermo Fisher Scientific S5 sequencing platform automatically performs a set of next generation sequencing reads and quality checks statistics pre- and post-alignment with the hg19 human reference genome. Statistics includes read length histograms, chip-loading-density percentage, total number of mapper and unmapped reads, and similar self-explanatory statistics. The most useful ones are the “on target”, “uniformity”, and “mean depth” statistics from the Thermo Fisher Scientific “coverage analysis” plugin. They represent the alignment statistics for each amplicon of the panel, where the “on target” statistic represents the percentage of reads aligning in correspondence of an amplicon included in the panel; the “uniformity” statistic represents the percentage of the amplicon bases covered by at least 0.2 × the average base reads depth; and the “mean depth” statistic represents the average base coverage depth over all bases targeted in the reference. To be evaluable, we expect samples to have “on target” value > 90%, “uniformity” value > 90%, and “mean depth” > 8000 for cffDNA samples (and > 400 for gDNA samples). The Thermo Fisher Scientific system produces a BAM file for each sample, containing all the aligned reads. This file is exportable from the system and can be submitted for further bioinformatics analysis. In this study, the BAM files obtained from the S5 instrument have been analyzed using the NIPAT-flow data analysis pipeline developed by the Eurofins Genoma Group.

### 2.2. NIPAT-Flow Algorithm

The algorithm evaluates the compatibility of each maternal cell-free fetal DNA sample against each alleged father. The fetal genotypes for each SNV included in the analysis are inferred from the maternal samples. Furthermore, the algorithm robustness has been validated using a set of mock samples generated by simulating 100 biological brothers for each biological father.

The algorithm utilizes the BAM files obtained from the next-generation sequencing process, and it produces some intermediate reports (one for each mother vs. alleged father comparison) and a final overall report including the paternity probability (W) for each comparison. The evaluation is straightforward from the Combined Paternity Index (CPI likelihood statistic) adapted to be used in the context of an SNV-based prenatal test.

A kinship relationship is universally evaluated by comparing the likelihoods of observing the obtained genotypes given two alternative hypotheses (i.e., the Likelihood Ratio, LR). In the case of paternity testing, it is evaluated whether an individual is related to another individual with a father–son relationship versus the hypothesis that the two individuals are not related. The higher the LR, the more supported is the first hypothesis (paternity). The lower the LR (i.e., <1), the more supported is the second hypothesis (unrelated individuals). For each SNV, the Paternity Index (PI) is classically calculated as a likelihood ratio according to the Bayesian theorem [22]. PI is defined as the ratio between the probability of the fetal genotype to be the observed one (event E) conditioned to the alleged father being the biological father (hypothesis H_1_) and the same probability where the father is a random individual extracted from the population (hypothesis H_2_) (PI = Pr(E | H_1_)/Pr(E | H_2_) [22]. When multiple loci are used to determine paternity, the product of all the individual PI values for each locus is the combined paternity index. PI formulas are adapted to the cases of prenatal tests where fetal genotypes need to be inferred from the maternal cffDNA samples [16,23,24]. In particular, this was undertaken by also taking into account technical errors and natural effects (e.g., sequencing errors or ex novo mutations) that could lead to fetal genotype misinterpretation and eventually to a biased PI calculation. The CPI for a couple is then the product of all the PIs—one for each SNV included in the analysis—and paternity probability (W) is calculated as (CPI/CPI + 1)*100.

Only SNVs where the mother genotype is homozygous have been included in CPI calculation because for heterozygous maternal positions it is statistically inaccurate to infer the fetal genotype from the maternal cell-free fetal DNA sample only [24,25]. 

The algorithm defines the fetal genotype from the maternal cffDNA sample using a set of fetal base thresholds. In fact, for each SNV, if a low-frequency base is detected on the maternal sample, and it is different from the maternal homozygous genotype, the fetal genotype is inferred as heterozygous. In particular, a minimal coverage of 1000 reads for the maternal sample and 100 reads for the alleged father are required for the inclusion in the Paternity Index calculations. A base coverage of at least 100 reads is required to be assigned as fetal for allele characterized by frequency ranging from 1.5% to 15%.

In the case of variants located on chromosome Y, the maternal zygosity filter is obviously inapplicable; however, for a coverage > 100 reads, the filter is still applied. In the end, the number of SNVs reporting a low-frequency allele varies among different meiosis samples, ranging from 131 to 173 with an average of 145 SNVs.

Multiple checks and features are included in the algorithm to improve the robustness against both human and technological errors. The algorithm performs an assessment of relatedness indexes among all different sample pairs [24,26]. Some thresholds are set in the algorithm to deal with noise and low coverage. In particular, the algorithm includes a noise reduction method to ensure a more robust call for the fetal base, starting from the mother’s genotype. Fetal genotype calling implements a SNV-specific threshold for the low-frequency alleles which relies on previously collected data of low-frequency alleles on samples without cffDNA. As a support, CPI is also calculated using a different set of thresholds optimized for low-fetal-fraction samples. Robustness of the CPI calculation is also assured by the usage of no less than 30 SNVs reporting a low-frequency allele (1.5–15%).

### 2.3. Simulating Father’s Brothers

For each compatible couple, 100 synthetic brothers of the designated father were simulated (for a total of 900 simulate samples) to evaluate the performances of NIPAT-flow on individuals whose genetic profile was closely related to the real biological father. A two-step probabilistic model was designed to define the synthetic sample’s genotype for each SNV. Each father’s brother is then a sample drawn from this model. Given the designated father, a couple of synthetic parents was first sampled from an inferred probability distribution and then a synthetic son of theirs was generated using the equiprobable combination of their genotypes. In more detail, a Bayesian approach was used to infer the parents’ genotypes. For each SNV, the vector of the MAFs values was taken as the prior probability distribution. This quantity was updated considering the profile of the designated father, using the likelihood of its genotype conditioned to his parents’. The normalized product of these two quantities is a posterior probability distribution from which the genotypes of the parents are sampled. This probabilistic model allows us to create individuals sharing a major part of their genetic profile with the designated father. Defining concordance between two individuals as the percentage of SNVs showing an identical genotype over the total number of SNVs, the brothers showed, on average, a concordance of 68.6% with the designated father. This percentage varies equally both across the different brothers and different individuals (64.0–72.8%). As expected, the concordance between unrelated individuals was lower, ranging from 18.9% to 56.4% (average 36.1%). 

### 2.4. Statistical Analysis

To evaluate the reliability and robustness of NIPAT, couples originating from the maternal samples and biological fathers (*n* = 9), unrelated fathers (*n* = 72), and simulated brothers (*n* = 900) were tested by comparing the log(CPI) distributions between groups.

At first, the log(CPI) parent distribution was tested for normality using a Shapiro–Wilk test and evaluated with a skewness–kurtosis plot for empirical distribution [27]. This plot combines information about skewness and kurtosis, which are measures of the shape of a distribution. A skewness–kurtosis plot can help decide whether a parametric or non-parametric statistical test is appropriate by determining whether the distribution of the data is normal or non-normal. Bootstrapping was used (nboot = 100) to test the stability of the skewness and kurtosis statistics when data were resampled, finally ensuring evaluation reliability. As the distribution of log(CPI) was identified as non-normal, a non-parametric statistical test was chosen, considering that the inappropriate use of a parametric test would have given biased results. A Kruskal–Wallis rank sum test was used to test if there was a difference in log(CPI) between biological fathers, unrelated couples, and simulated brothers. Post hoc tests were then performed with the two-tailed Wilcoxon test for two independent samples and p-values were corrected for multiple testing using the false discovery rate correction. We set α = 0.0001, corresponding to a confidence level (1 − α) of 99.99%. This highly restrictive confidence level was set to reduce the risk of incurring a false positive result. Moreover, to further confirm our result, we decided to compute a 99.99% confidence interval for the true mean of the simulated brothers log(CPI) distribution. The statistical analysis was performed with the R programming language [28].

## 3. Results

Here, we present a non-invasive prenatal paternity test (NIPAT) using cffDNA based on Ion S5 NGS technology. A custom PCR amplification panel consisting of 861 SNVs has been developed on the basis of MAF and the absence of correlation with human phenotypes. A number of nine pregnant women and their partners were recruited to test the performance of NIPAT. In particular, maternal peripheral blood samples were utilized for the extraction of cell-free fetal DNA as a source of fetal material for the NIPAT workflow. Informative SNVs used for CPI calculations were selected based on the mother genotype. The number of SNVs reporting a maternal homozygous genotype and a second low-frequency allele in our cohort ranges from 131 to 173, with an average of 145 SNVs. 

Log(CPI) values calculated for designated fathers showed ranges between +34 and +85, whereas log(CPI) values calculated for unrelated individuals were below −150 (full data available in Supplementary Table S1). In order to evaluate the performances of NIPAT on individuals whose genetic profile was closely related to the biological father, for each compatible couple, 100 synthetic full brothers of the designated father were simulated (for a total of 900 simulate samples).

Log(CPI) values calculated for simulated full brothers of the designated fathers ranged between −30 and −200 with an average of −108 (Supplementary Figure S1). These values are still very far from the biological father log(CPI) value. These data truly support the robustness of the NIPAT-flow test. 

Finally, the performances of NIPAT were fairly concordant with the paternity compatibility threshold (log(CPI) > + 4) and paternity exclusion threshold (log(CPI) < −4) suggested by the reference guidelines and reference literature for SNV approaches [21,26].

**Figure 1:**
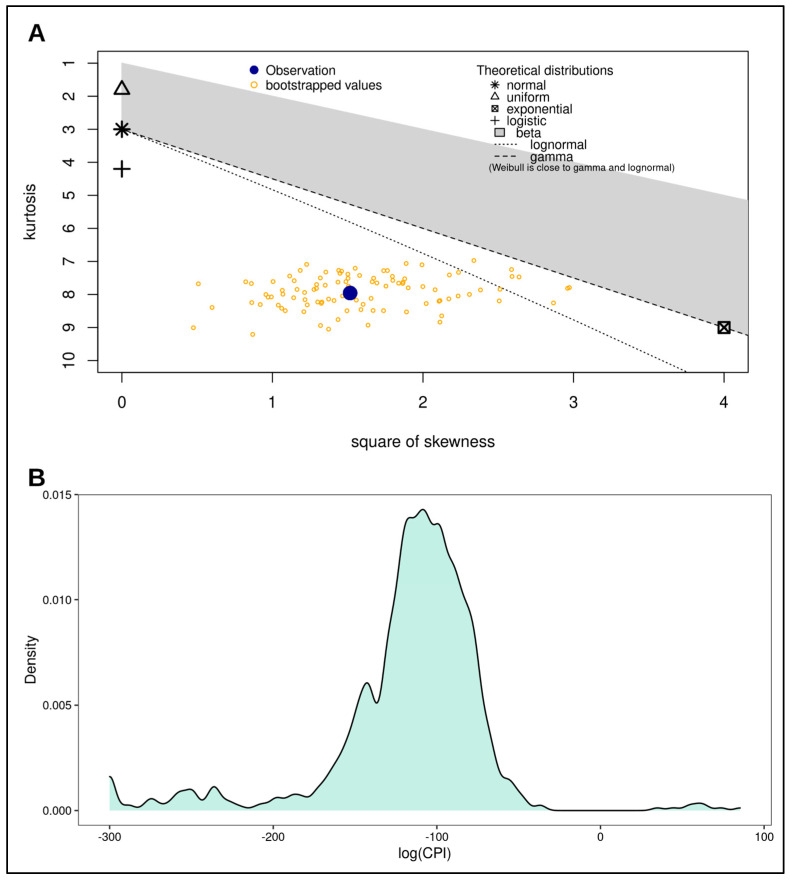
Distribution assessment for log(CPI). (**A**) Skewness-Kurtosis plot of log(CPI). This plot was produced as a further contribution for the assessment of normality for the log(CPI) distribution after the Shapiro-Wilk test. This plot shows the distribution of log(CPI) as determined by square of skewness and kurtosis. The blue dot represents statistics for the distribution, while the yellow dots represent the statistics for the bootstrapped distribution. Based on the position of the blue dot with respect to the position of the theoretical distributions, it is clear to see that both the blue dot and the yellow dots are far from the normal distribution. This indicates the distribution of log(CPI) is non-normal. (**B**) Density curve of log(CPI) highlighting the shape of the distribution. This plot was produced as a visual assessment of log(CPI) distribution, which is non-normal.

To assess the differences between the distributions of log(CPI) resulting from real fathers, unrelated couples, and simulated full brothers, we first evaluated the parent distribution of log(CPI). A Shapiro–Wilk normality test showed that the distribution was strongly non-normal (W = 0.83711, *p*-value < 0.0001), as also shown in the skewness–kurtosis plot, and noticeable in the density plot (Figure 1). Therefore, we used a non-parametric approach to hypothesis testing.

**Figure 2:**
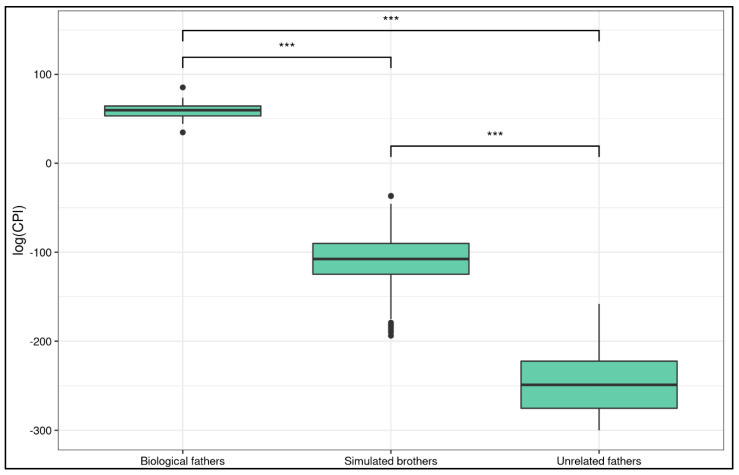
Log(CPI) distribution in different groups. The boxplot shows the comparison among biological fathers, simulated brothers and unrelated fathers. Stars report Wilcoxon test *p*-value (*** is *p* ≤ 1.146 × 10^−6^).

A Kruskal–Wallis rank sum test showed that there was a statistically significant difference in log(CPI) between real fathers, unrelated couples, and simulated full brothers (χ^2^ = 221.55, df = 2, *p*-value < 0.0001). Post hoc Wilcoxon tests reported a significant difference in all the comparisons (Figure 2).

The log(CPI) of the simulated brothers was significantly different from that of the biological fathers (W = 8100, *p*-value = 2.38 × 10^−7^, *p*-value adjusted = 3.57 × 10^−7^, 99.99% CI [−203.3075, −134.5006]). Both the lower and upper CI bounds were negative, meaning that the true mean of the simulated brothers cannot overlap with that of the biological fathers. In fact, the estimated difference in location of the means is 166.6658. The log(CPI) of the unrelated couples was significantly different from that of the biological fathers (W = 648, *p*-value = 1.146 × 10^−6^, *p*-value adjusted = 1.146 × 10^−6^, 99.99% CI [−359.8096, −243.4905]). The log(CPI) of the simulated brothers was significantly different from that of the unrelated couples (W = 64691, *p*-value = 4.559 × 10^−45^, *p*-value adjusted = 1.367 × 10^−44^, 99.99% CI [−156.0940, −121.1659]). 

As the NIPAT-flow algorithm reported zero chances of obtaining a log(CPI) value greater than 0 for the simulated brothers and for the unrelated couples, we conclude that it can be used to flawlessly identify biological fathers. Such a high statistical significance in testing biological father vs. simulated brothers and vs. unrelated couples’ log(CPI) values ensures that NIPAT is flawlessly reliable in detecting the biological father.

## 4. Discussion

Here, we present a Non-Invasive Prenatal Paternity Test (NIPAT) using cffDNA based on Ion S5 NGS technology that is already used in many laboratories for forensic NGS protocols. A custom PCR amplification panel consisting of 861 SNVs has been developed on the basis of MAF. The average MAF of the panel is 0.321 (median in 0.347), while the number of SNVs per chromosome ranges from 12 to 95 (with an average of 36). The algorithm was tested on a number of nine pregnant women and their partners. NIPAT generated log(CPI) values for designated fathers ranging from +34 to +85, whereas log(CPI) values calculated for unrelated individuals were less than −150. This difference in log(CPI) values between these two groups demonstrates the robustness of NIPAT, making it an extremely reliable tool for determining paternity with a high degree of confidence. 

One of the main challenges for paternity testing is the ability to distinguish two possible fathers when they are biologically related. It is not particularly rare that two possible fathers can be related and, therefore, share many DNA variants. Thus, getting conclusive results for a paternity test may be challenging using traditional short tandem repeats (STR)-based methods. Full brothers share 50% of their DNA and represent a typical case of disputed paternity between related putative fathers. To evaluate the robustness of NIPAT, we calculate log(CPI) for 100 simulated full brothers of each biological father. More than 900 meiosis samples were analyzed, and log(CPI) values were compared between biological fathers and 100 virtual full brothers. Log(CPI) values were calculated for simulated full brothers of the designated fathers, ranging between −30 and −200 with an average of −108. The difference between log(CPI) values for designated fathers and simulated full brothers was very high, and these two distributions never overlapped. Thus, this means that the chance of incurring a false positive is approximately 0, meaning that the NIPAT test is robust with high rates of shared DNA. 

Finally, the performances of NIPAT are fairly concordant with the paternity compatibility threshold log(CPI) > + 4 and the paternity exclusion threshold log(CPI) < −4 suggested by reference guidelines and reference literature for SNV approaches [21,26]. To date, there are no universally accepted thresholds for the confirmation of paternity, exclusion, or for inconclusive results for both STRs- and NGS-based methods. Accredited laboratories are expected to establish an internal range for inconclusive results, with such values dependent on the methods, the validation studies, the number of SNPs, etc. We would like to outline that NIPAT showed differences in terms of log(CPI) between designated fathers and simulated full brothers that were very striking and unimaginable with conventional analysis. Generally accepted ranges for inconclusive cases are 10^−2^ < LR < 10^2^, or 10^−4^ < LR < 10^4^ [29]. The CPI values calculated for designated fathers ranged between 10^+34^ and 10^+85^, whereas the CPI values calculated for unrelated individuals were below 10^−150^. As a stress test, CPI values calculated for simulated full brothers of the designated father ranged between 10^−30^ and 10^−200^, with an average of 10^−108^.

These data confirm that a genomic approach, analyzing hundreds of variants based on next-generation sequencing, can represent an opportunity for paternity testing compared to traditional methods based on STR typing. The ability to interpret the sequence of hundreds/thousands of SNVs allows discriminating powers unimaginable only a few years ago. Non-invasive prenatal paternity tests using cell-free fetal DNA need to analyze hundreds of genetic variants, and NGS technology and statistical approaches are mature enough to support robust methods ensuring the correctness of results. We believe that these data strongly support the robustness of the NIPAT-flow test, representing an interesting approach for scientists working in the field. 

## Data Availability

All data generated in this manuscript are included within the manuscript.

## Supplementary Materials

The following supporting information can be downloaded at: https://www.mdpi.com/article/10.3390/genes14020312/s1. Figure S1: Log(CPI) comparison; Table S1: Log(CPI) values.
