# Supplementary material for: NIPAT as Non-Invasive Prenatal Paternity Testing Using a Panel of 861 SNVs
Source: Genes (Basel). 2023 Jan 25;14(2):312. doi: 10.3390/genes14020312 (PMC9957069; doi:10.3390/genes14020312)
Supplement: Supplementary file 1 [file genes-14-00312-s001.zip › Supplementary Materials/Supplementary Figure S1.pptx]

## Slide 1
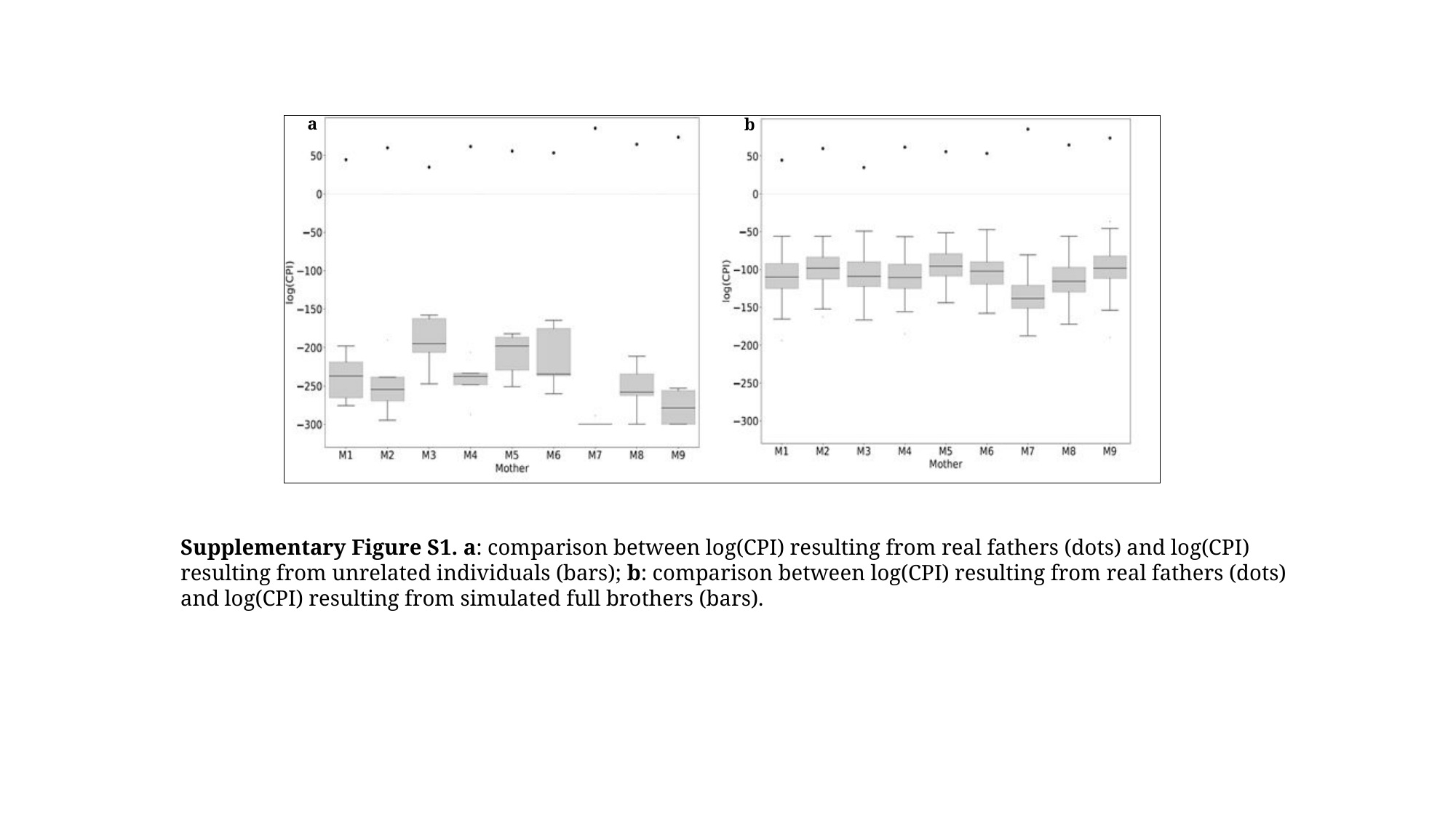

a
b
Supplementary Figure S1. a: comparison between log(CPI) resulting from real fathers (dots) and log(CPI) resulting from unrelated individuals (bars); b: comparison between log(CPI) resulting from real fathers (dots) and log(CPI) resulting from simulated full brothers (bars).
